# Diabetes self-management in online health communities: an information exchange perspective

**DOI:** 10.1186/s12911-021-01561-3

**Published:** 2021-06-28

**Authors:** Jing Min, Yan Chen, Li Wang, Ting He, Sha Tang

**Affiliations:** Department of Endocrinology, The Central Hospital of Wuhan, No. 26, Shengli Street, Jiang’an District, Wuhan, 430000 Hubei Province China

**Keywords:** eHealth, Health Information Exchange, Self-management, Health care costs

## Abstract

**Background:**

Online health communities (OHCs), with a wealth of multi-source information exchange, have provided a convenient way for people with diabetes to actively participate in their self-management and have been widely used. Information exchange assists people with diabetes with health-related decisions to actively engage in their care, and reduce the occurrence of potential complications of diabetes. However, there has been relatively little research on the information exchange behaviors and their effect on health in professional online medical platforms—OHCs.

**Objective:**

Using a social exchange theory, this study focuses on two sources of information (doctors and people with diabetes) to investigate information exchange behaviors and consequences. Moreover, we also examine moderating effects of information price as patients need to pay prices for consulting with doctors to obtain medical information on OHCs.

**Methods:**

By using the Python program, a rich dataset contained 22,746 doctor-patient dialogues from December 2017 to December 2018 is collected from the biggest OHC in China. Then the logistic and ordinal regression models are used to get empirical results.

**Results:**

We found that first information sharing from doctors and other people with diabetes can promote their information sharing behavior. Second, the moderating effects of information price are heterogeneous and change with the exchange participants. Third, rich information exchange supports self-management of people with diabetes and improves their health status.

**Conclusion:**

This study is among the first that tests the information exchange behavior and consequence for diabetes in OHCs and examines the moderating effects of the information price. The present study produces several insights, which have implications for social exchange, patient behavior, online health communities, and information technology in diabetes self-management literature.

## Background

Diabetes is one of the major chronic non-communicable diseases in the world [1]. With the increasing prevalence rate in recent years, the disease burden brought by diabetes has become increasingly serious, and diabetes and its complications have seriously affected the quality of life [2]. In 2018, there were nearly 129.8 million people in China suffered from diabetes with 20.2% in people aged 18 to 29 and more than 40% in people aged ≥ 40 [3]. The prevalence of diabetes continues to rise without a plateau or inflection point. More threateningly, people lack awareness of diseases, with the awareness rate is only 30.1% in China [1].

Diabetes is a metabolic disorder caused by genetic and environmental factors, and these potential complications of diabetes can be controlled by lifestyle management [4, 5]. For example, people with diabetes who are compliant with their regimen and maintain strict glycemic control have lower rates of complications [6]. Different from other diseases, access to information can provide people with diabetes the tools and support to self-manage their condition effectively [4, 5], as this chronic disease changes over time. However, in the traditional medical environment, there is a lack of information provided to people after their formal diagnosis [7]. Online platforms, with a wealth of multi-source information exchange, have provided a convenient way for diabetes to access information and have been widely used.

Online health communities (OHCs), are different from general communities, provide an important platform for people to obtain information and learn from others with similar experiences [8], and have developed rapidly around the world. Multi-type services, various forums, multi-participants, and feedback mechanisms on OHCs offer people the opportunity to manage their condition of diseases by exchanging information. OHCs have been the focus of considerable attention as the number of patients relying on online health information has steadily increased. OHCs include access to alternate sources of health information as well as a way to connect with others with similar diseases. Information on OHCs has been found to affect patients’ decision-making and participation in healthcare [9, 10].

By reviewing the literature on diabetes, information exchange, and OHCs, three important research gaps are found. First, although online social support has proved to improve health behaviors and decrease symptoms [11], prior studies mainly used survey design and focused on general online social media (e.g. Facebook and Twitter) [12]. Hence, evidence of the effects of professional online medical platforms—OHCs for diabetes is lacking. Second, although information exchange on OHCs has received research attention [13–16], they failed to consider information exchange among multi-participants in one study and investigate their integrated effects. Third, more importantly, no study has explored the role of information cost (i.e. the costs for obtaining information on diseases) in information exchange.

Urbanization, the aging population, reduced physical activities, and increasing levels of overweight and obesity contribute to the increase of diabetes. OHCs, supplement traditional services in hospitals, create information-rich environments that allow people with diabetes to seek and share information effectively and improve their ability to self-manage. To fill gaps in existing studies, this study focuses on two forms of exchange (i.e., exchange between doctors and diabetic patients, and exchange between diabetic patients and diabetic patients) to investigate information exchange behaviors and consequences by collecting a real operational dataset from a famous OHC in China. As people need to pay prices for consulting with doctors to obtain medical information on OHCs, we also examine moderating effects of information price. The specific research questions being addressed in this paper are:

1. Does online information sharing from a doctor affect a diabetic patient’s propensity to share information publicly?
2. Does online information sharing from other patients affect a diabetic patient’s propensity to share information publicly?
3. How does information price moderate the information exchange behaviors on OHCs?
4. Does information exchange in OHCs affect the health status of people with diabetes?

By understanding the online information exchange behaviors of doctor-patient and patient-patient, we may be able to understand how to reach people to receive and deliver diabetes information through these professional OHCs, and finally manage their health effectively.

This study is organized as follows: We begin by reviewing the theoretical background and developing hypotheses. The next section presents research methods. The empirical results are provided in the next section. We then conclude and discuss the contributions, implications, and limitations of this study.

## Hypotheses development

### The social exchange theory

The social exchange theory seeks to explain individual behavior in the process of resource exchange [17] and assumes the existence of relatively long-term relationships of interest as opposed to one-off exchanges [18]. It has been widely used to explore individual behavior in the online environment [10, 19, 20]. Based on the social exchange theory, individuals engage in social interaction in the hope of getting returns back from the interaction. Information exchange in virtual communities is a reciprocal process with maximizing benefits and minimizing costs. The social exchange theory has been applied to study consumers’ motivation to provide reviews online [10].

Benefits in social exchange have been defined as exchange outcomes that provide positive values [18]. Intrinsic benefits and extrinsic benefits determine users’ exchange behavior together [21]. The intrinsic benefits are an experience of pleasure and satisfaction inherent in the exchange activity, and extrinsic benefits focus on outcomes that may be obtained as a result of the behavior rather than engaging in it for its inherent satisfaction [22]. Costs in the social exchange are defined as negative outcomes from exchanges, including intrinsic cost and opportunity cost [18]. Applying the social exchange theory, we expect that people with diabetes are motivated to share information if they are satisfied with the benefits received based on information sharing from others in OHCs.

### Information exchange in OHCs

From the information exchange perspective, users participate in virtual communities in two ways: information seeking and information sharing [23], which are indispensable parts of virtual communities [23, 24]. In general virtual communities, users can seek information by browsing or posting questions, and also sharing information by participating or initiating in forums or replying to questions. The sustained provision of information concerns the long-term development of the virtual communities and in turn, affects their users’ benefits. During the information exchange process, users can receive not only information but also social support within communities, which enhances their sense of communities [25] and motivates their active participation in virtual communities.

There are three types of information exchange that exist on OHCs: doctor-patient, patient-patient, and doctor-doctor. Information on OHCs has been found to affect patients’ decision-making and participation in healthcare [7, 10]. However, although information exchange on OHCs has received research attention [13–16], they failed to consider information exchange among multi-participants in one study and investigate their integrated effects. In addition, although online social supports have proved to have effects on improving health behaviors and decreasing symptoms [26], prior studies mainly used survey design and focus on general online social media (e.g. Facebook and Twitter) [12], evidence of the effects of professional online medical platforms—OHCs for diabetes is lacking.

Information sharing includes exchanges of information with multiple parties that may be useful to everyone [27] and is completely voluntary. In OHCs, feedback from patients includes important information on doctors’ services, which can influence service evaluations and purchase decisions [7]. Feedback has multiple forms, such as detailed reviews, short comments and ratings. As detailed reviews contain richer information than short comments or ratings do, we focus on detailed review sharing behavior in this study. Based on the social exchange theory, when users have received information from others, they have received benefits and tend to share information with others. Information mainly comes from two sources, namely doctors and diabetic patients. Hence, we have:

- **H1a.** The information sharing from a doctor has a positive impact on a diabetic patient’s information sharing behavior in OHCs.
- **H1b.** The information sharing from other patients has a positive impact on a diabetic patient’s information sharing behavior in OHCs.
- **H2.** Diabetic patients who have engaged in information exchange will have a better health status.

### Information cost as a moderating factor

The motivation crowding theory suggests that users’ motivations to exchange can be undermined or strengthened under different conditions [28]. Except for the benefits, consumers’ perceived cost is the core construct and foundation in an exchange [29] and may impede consumers to engage in an exchange [30]. Price has played a moderating role in the exchange between doctors and patients [10].

On the one hand, price is the cost for obtaining information. Transaction cost economics [31] tells us that transaction costs will reduce purchase decisions. When patients have paid higher prices to obtain medical information, they may have lower a propensity to share relative information with others. On the other hand, although receiving information from others helps patients to gain knowledge on both diseases and doctors, if a patient still wants to buy services from doctors, it means that the current information does not provide enough knowledge to him. Then the information price is what he or she gives up to acquire and use a service. Hence, we have:

- **H3a:** Information price negatively moderates the relationship between information sharing from a doctor and a diabetic patient’s information sharing behavior in OHCs.
- **H3b:** Information price negatively moderates the relationship between information sharing from other diabetic patients and a diabetic patient’s information sharing behavior in OHCs.

**Fig. 1:**
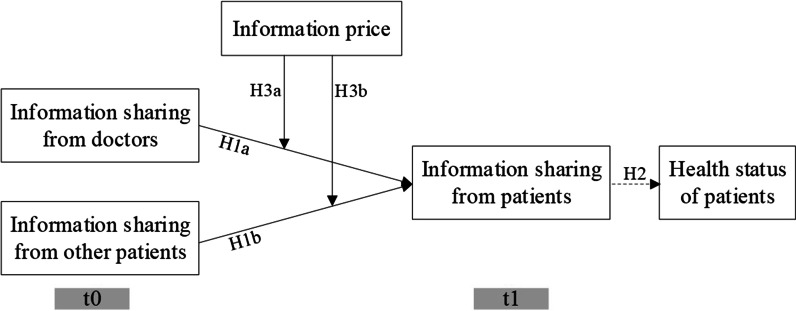
The conceptual model

Figure 1 shows our conceptual research model.

## Methods

### Research context

We collected data from the biggest OHC in China—Haodf.com (www.haodf.com), founded in 2006. Haodf.com offers hospital/doctor information inquiry, text consultation, telephone consultation, remote video outpatient service, accurate outpatient appointment, disease management after diagnosis, family doctor, science popularization of disease knowledge and other fields, and has been widely trusted by doctors and patients.

Haodf has collected the information of 610,000 doctors from 9917 hospitals in China by December 2019. 230,000 doctors have registered with their real names on the platform and began providing online medical services to patients. The proportion of doctors from tertiary hospitals accounts for 78%. Haodf has served more than 58 million patients. Haodf presents a list of doctors based on diseases; 30,272 diabetes-related information exchanges have occurred, so that understanding the information exchange behavior in this context will help us understand and further improve the healthcare industry.

On Haodf, text consultation is the most popular service among patients. As a doctor can price his services on Haodf, it is an important platform for doctors to provide paid services. At the same time, Haodf also provides doctors with choices to provide free text consultation services (Note: in our paper, we call free information and paid information for information obtained from the free text consultation service and paid text consultation service, respectively.) Although the information is shared voluntarily and can be accessed by all users, the information shared from patients is based on the paid service and the information shared from doctors can also be charged but they choose not to. This means that it is priced as a paid service but given free. Therefore, the value of information shared by patients and doctors can be measured by the doctor’s service price (Note: we call service price as information price.) Haodf enabled us to comprehensively understand the free information exchange behavior and examine the moderating effects of information price.

### Sample and data collection

**Fig. 2:**
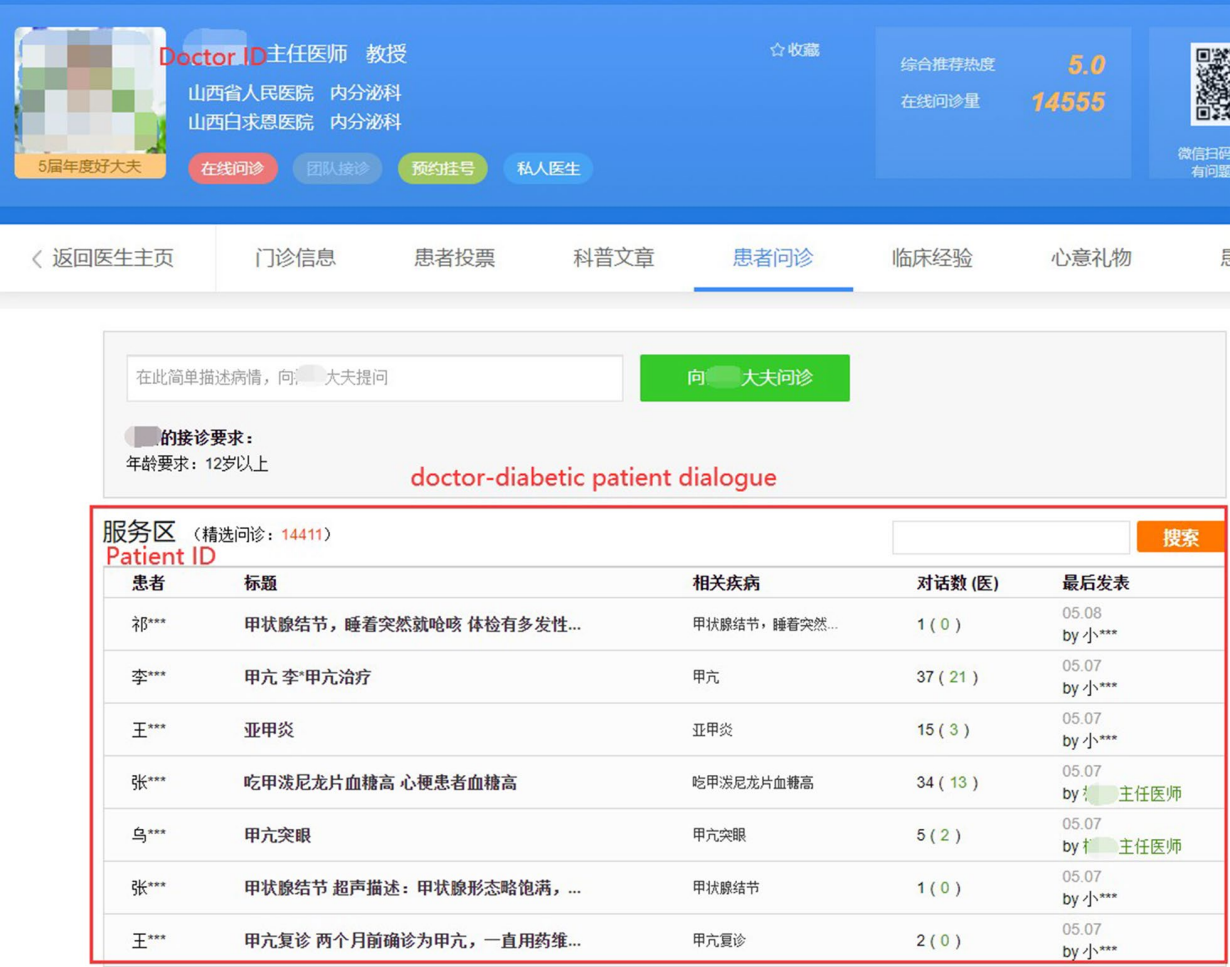
A doctor’s homepage

This study quantitatively explores the information exchange behavior and its consequence on health status in the online environment. On Haodf, all interactions between doctors and patients are recorded on the doctor’s homepage (See Fig. 2). Based on the list of diabetes doctors, we included these diabetes doctors who have provided the text consultation service and used the platform within three days to ensure the doctor was active. We deleted those doctors missing important information, such as service price, yielding a sample of 2,028 doctors. Then we collected all information exchanges on diabetes from doctors’ homepages from December 2017 to December 2018 by using the Python program. The collecting process lasted one week with 22,746 doctor-diabetic patient dialogues included. Besides the detailed doctor-diabetic patient dialogues, we also collected doctors’ individual information, including medical title, education title, gender, educational background, popularity on Haodf, years of experience on Haodf, service quality, level of hospital and economic level of the city where the doctor works.

### Variables and models

As we have collected all doctor-diabetic patient dialogues, we can get the provided time for each interaction. Information from doctors and other diabetic patients (t0) is provided earlier than the information from diabetic patients (t1).

**Dependent variables** include (a) information sharing from diabetic patient *i* (*ISP*) and (b) the health status of diabetic patient *i* (*HSP*). Based on a doctor-diabetic patient dialogue, we can get whether diabetic patient *i* has shared his feedback on information received from doctor *j* and also get his health status based on the feedback. A dummy variable (*ISP*) is set to measure whether diabetic patient *i* has shared information after receiving information from doctor *j*. There are four main health statuses (cured, better, no better, worse) for a diabetic patient to choose by answering a questionnaire on Haodf. We use an ordinal variable (*HSP*) to measure the health status of diabetic patient *i*, with a larger value indicates a better status.

**Independent variables** include (a) information sharing from doctor *j* (*ISD*) and (b) information sharing from other people with diabetes (*ISOP*) of doctor *j*. For the information sharing from doctor *j*, as we only include free information sharing behavior, we distinguished whether doctor *j* has provided free service for diabetic patient *i* based on the doctor-diabetic patient dialogue. A dummy variable (*ISD*) is used to measure information sharing from doctor *j*. For the information sharing from other people with diabetes of doctor *j*, we calculated the number of information sharing from other people with diabetes (*ISOP*) of doctor *j*, which happened before the produced time of information sharing from diabetic patient *i*.

**The moderating variable** is the information price (*IP*). A diabetic patient needs to pay a service fee for getting a doctor’s service to obtain relevant information on diseases. Therefore, the information price is measured based on the service price of doctor *j* in our model.

**Control variables** are medical title, education title, gender, educational background, popularity on Haodf, experience on Haodf, response speed, level of hospital, and economic level of the city where the doctor works. Two dummy variables (*MTitle1* and *MTtitle2*) are used to measure medical title (chief doctor, associate chief doctor, others). A dummy variable (*ETitle*) is set to measure education title (professor/associate professor, others). A dummy variable (*Education*) is used to measure whether the highest degree of the doctor is obtained from a foreign university. On Haodf, a “recommendation” is calculated by the website to measure a doctor’s activities and his patients’ ratings, which is used to measure the popularity of the doctor in our study (*Recommendation*). The number of days (*Days*) that the doctor has joined Haodf is used to measure the experience on Haodf. Response speed (*RR*) measures the time a patient waits for a doctor’s reply. A dummy variable (*Level*) is used to measure the level of the hospital (tertiary hospitals and others) that the doctor works in. As the economic level of the city where the doctor works may influence the doctor’s pricing and patients’ consumption ability, a dummy variable (*City*) is set to measure whether the doctor comes from top-tier cities (Beijing, Shanghai, Guangzhou and Shenzhen).

**Model estimation.** The empirical analysis is conducted into two steps. We first analyze the information exchange behavior and moderating effects of information price, and then investigate the impact of information sharing from the doctor and other diabetic patients on the diabetic patient’s health status. The regression models are as follows:

$$\begin{aligned} ISP_{{ij}} & = \beta _{0} + \beta _{1} ISD_{{ij}} + \beta _{2} ISOP_{{ij}} + \beta _{3} IP_{j} + \beta _{4} ISD_{{ij}} \times IP_{j} + \beta _{5} ISOP_{{ij}} \times IP_{j} \\ & \quad + \beta _{6} MTitle1_{j} + \beta _{7} MTitle2_{j} + \beta _{8} ETitle_{j} + \beta _{9} Education_{j} \\ & \quad + \beta _{{10}} Recommendation_{j} + \beta _{{11}} Days_{j} + \beta _{{12}} RR_{{ij}} \\ & \quad + \beta _{{13}} Level_{j} + \beta _{{14}} City_{j} + \varepsilon _{i} \\ \end{aligned}$$

$$\begin{aligned} HSP_{{ij}} & = \beta _{0} + \beta _{1} ISD_{{ij}} + \beta _{2} ISOP_{{ij}} + \beta _{6} MTitle1_{j} + \beta _{7} MTitle2_{j} \\ & \quad + \beta _{8} ETitle_{j} + \beta _{9} Education_{j} + \beta _{{10}} Recommendation_{j} \\ & \quad + \beta _{{11}} Days_{j} + \beta _{{12}} RR_{{ij}} + \beta _{{13}} Level_{j} + \beta _{{14}} City_{j} + \varepsilon _{i} \\ \end{aligned}$$

where *i* = 1, …, n presents the diabetic patient and *j* = 1, …, n presents the doctor. *ISD*_*ij*_ × *IP*_*j*_, *ISOP*_*ij*_ × *IP*_*j*_ are interaction effects.* β* are coefficients needed to be estimated, and *ε* is the error.

## Results

### Hypotheses testing

The logistic and ordinal regression models are used to get our empirical results by using STATA. Table 1 shows the descriptive statistics and correlations of the variables. Tables 2 and 3 present the empirical results.

**Table 1:** Descriptive statistics and correlations

	Mean	SD	1	2	3	4	5	6	7	8	9	10	11	12	13	14
1. HSP	2.517	1.634														
2. ISP	0.335	0.472	0.440**													
3. ISD	0.297	0.770	0.706**	0.565**												
4. ISOP	15.819	39.507	0.153**	0.016	0.053**											
5. IP	62.529	59.821	0.046	0.035	0.067	0.016										
6. MTitle1	–	–	0.050*	0.023	0.053*	0.009	0.570									
7. MTitle2	–	–	0.372*	0.372*	0.441*	0.171*	0.162*	0.342*								
8. ETitle	–	–	0.136	0.215	0.186*	0.015	0.020	0.008	0.169*							
9. Gender	–	–	0.058*	0.009	0.053*	0.074	0.050	0.042*	0.136	0.046						
10. Education	–	–	0.438	0.496	0.510	0.070	0.056	0.071	0.532	0.110	0.224					
11. Recommendation	4.044	0.327	0.007	0.147**	0.030*	0.078**	0.035*	0.007	0.080**	0.007	0.025	0.438*				
12. Days	1121.400	2364.203	0.438**	0.496**	0.510**	0.070**	0.056**	0.071*	0.532**	0.110**	0.303**	0.238**	0.163			
13. RR	0.699	0.322	0.224**	0.177**	0.268**	0.104**	− 0.215	− 0.017	0.399**	0.006	0.078**	0.09**	0.303**	0.257		
14. Level	–	–	0.022	0.074**	0.031*	0.058**	0.253	− 0.058	0.019	0.007	0.062	0.023	0.145	− 0.131	0.067	
15. City	–	–	0.052*	0.016	0.196**	− 0.110*	− 0.034*	0.139**	0.052**	0.016	0.196**	0.238**	0.224	− 0.053	0.056	0.022

These numbers in the first row refer to the corresponding variables in the first column. ISP, information sharing from the diabetic patient; HSP, the health status of the diabetic patient; ISD, information sharing from doctor j; ISOP, information sharing from other people with diabetes; IP, information price

*p < 0.05; **p < 0.01; ***p < 0.001

**Table 2:** Results for information sharing from people with diabetes

Variables	Model 1	Model 2	Model 3
*ISD*		0.014*** (0.001)	0.016*** (0.003)
*ISOP*		0.009** (0.003)	0.004* (0.002)
*IP*		− 0.007* (0.003)	− 0.006 (0.004)
*ISD* × *IP*			− 0.005*** (0.001)
*ISOP* × *IP*			0.003* (0.001)
*MTitle1*	0.004 (0.005)	0.005 (0.005)	0.005 (0.005)
*MTitle2*	0.005 (0.005)	0.007 (0.005)	0.007 (0.005)
*ETitle*	0.009* (0.005)	0.008* (0.005)	0.008 (0.005)
*Gender*	− 0.004 (0.003)	− 0.003 (0.003)	− 0.002 (0.003)
*Education*	− 0.001 (0.004)	0.000 (0.004)	0.000 (0.004)
*Recommendation*	− 0.067*** (0.008)	− 0.040*** (0.009)	− 0.037** (0.009)
*RR*	0.000** (0.000)	0.001* (0.000)	0.002* (0.001)
*Days*	0.008** (0.002)	0.005* (0.003)	0.005 (0.003)
*Level1*	0.014* (0.006)	0.008 (0.006)	0.007 (0.006)
*City*	0.014** (0.004)	0.011** (0.004)	0.011* (0.004)
Adjusted R^2^	0.050	0.131	0.154
Sig. F change	0.000	0.000	0.000

ISP, information sharing from the diabetic patient; HSP, the health status of the diabetic patient; ISD, information sharing from doctor j; ISOP, information sharing from other people with diabetes; IP, information price

*p < 0.05; **p < 0.01; ***p < 0.001

**Table 3:** Results for health status of people with diabetes

Variables	Model 1	Model 2
*ISD*		0.009*** (0.001)
*ISOP*		0.018*** (0.003)
*MTitle1*	− 0.002 (0.003)	− 0.001 (0.003)
*MTitle2*	0.003 (0.003)	0.003 (0.003)
*ETitle*	− 0.004 (0.003)	− 0.002 (0.003)
*Gender*	0.006** (0.002)	0.004* (0.002)
*Education*_*i*_	0.004 (0.003)	0.005* (0.002)
*Recommendation*	− 0.009 (0.006)	− 0.017** (0.006)
*RR*	0.002*** (0.000)	0.007** (0.002)
*Days*	0.010*** (0.001)	− 0.000 (0.002)
*Level*	0.006 (0.004)	0.017 (0.006)
*City*	− 0.001 (0.003)	− 0.002 (0.003)
Adjusted R^2^	0.052	0.133
Sig. F change	0.000	0.000

ISP, information sharing from the diabetic patient; HSP, the health status of the diabetic patient; ISD, information sharing from doctor j; ISOP, information sharing from other people with diabetes; IP, information price

*p < 0.05; **p < 0.01; ***p < 0.001

From Table 1, we found that the mean of diabetic patients’ health status is 2.517, namely the status is between no better and better. 33.5% of diabetic patients choose to share information to others after receiving information from doctors and 29.7% of doctors have provided free service for patients. Based on the variables’ correlations, we found independent variables are significantly correlated with dependent variables.

From the results for information sharing from diabetic patients in Table 2, we found that both information sharing from the doctor (*β* = 0.014, *p* < 0.001) and information sharing from other diabetic patients (*β* = 0.009, *p* < 0.01) positively impact a diabetic patient’s information sharing behavior, and thus H1a and H1b are supported. The information price negatively moderates the relationship between information sharing from the doctor and a diabetic patient’s information sharing behavior (*β* = -0.005, *p* < 0.001), whereas information price positively moderates the relationship between information sharing from other diabetic patients and a diabetic patient’s information sharing behavior (*β* = 0.003, *p* < 0.05). Therefore, H3a is supported and H3b is not supported.

From the results of the health status of people with diabetes who have participated in information exchange in Table 3, we found that people with diabetes who have received information sharing from the doctor (*β* = 0.009, *p* < 0.001) and information sharing from other diabetic patients (*β* = 0.018, *p* < 0.003) have a better health status. Therefore, H2 is supported.

### Robustness check

In this section, we conducted another empirical analysis to ensure that our main results are robust. As people with diabetes can also get doctor’s services in the hospitals where the doctor work, which can influence a diabetic patient’s information exchange behavior online. Therefore, we deleted the samples of diabetic patients who had been seen in the doctors offline based on the doctor-diabetic patient dialogue and used the remaining subsamples to get empirical results (see Table 4). All the results in Table 4 were consistent with the main results. The empirical evidence further confirms the robustness of the information exchange in the online environment.

**Table 4:** Robustness check results

	Information sharing from people with diabetes	Health status of people with diabetes	
*ISD*	0.005*** (0.001)	0.007*** (0.003)	0.010*** (0.001)
*ISOP*	0.002* (0.001)	0.003* (0.002)	0.004*** (0.001)
*IP*	− 0.009*** (0.002)	− 0.003 (0.004)	
*ISD* × *IP*		− 0.006*** (0.001)	
*ISOP* × *IP*		0.004* (0.002)	
Adjusted R^2^	0.135	0.195	0.150
Sig. F change	0.000	0.000	0.000

ISP, information sharing from the diabetic patient; HSP, the health status of the diabetic patient; ISD, information sharing from doctor j; ISOP, information sharing from other people with diabetes; IP, information price

*p < 0.05; **p < 0.01; ***p < 0.001

## Discussion and implications

### Results analysis

To the best of our knowledge, our study is among the first that tests the information exchange behavior and consequence for diabetes in OHCs and examines the moderating effects of the information price. Our findings can help people understand the role of information exchange and OHC in self-management.

The results in Table 5 provide us with significant insight into the information exchange behavior and consequence in OHCs. Our research findings indicate that most of our hypotheses are supported. First, regarding the information exchange behavior on OHCs, this study demonstrated that receiving information from both doctors and other diabetic patients can enhance a diabetic patient’s propensity to share his treatment information publicly. These findings confirm the existence of social exchange behavior in the online environment, which is consistent with prior studies [10, 19, 20]. Doctors can gain diabetic patients’ feedbacks by providing free text consultation services.

**Table 5:** Summary of hypotheses

Hypotheses	Content	Supported or Not supported
H1a	The information sharing from a doctor has a positive impact on a diabetic patient’s information sharing behavior in OHCs	Supported
H1b	The information sharing from other diabetic patients has a positive impact on a diabetic patient’s information sharing behavior in OHCs	Supported
H2	Diabetic patients who have engaged in information exchange will have a better health status	Supported
H3a	Information price negatively moderates the relationship between information sharing from a doctor and a diabetic patient’s information sharing behavior in OHCs	No supported with opposite direction
H3b	Information price negatively moderates the relationship between information sharing from other diabetic patients and a diabetic patient’s information sharing behavior in OHCs	Supported

Second, information exchange behavior has a positive effect on the diabetic patient’s health status. The results suggest that when a diabetic patient has participated in online information exchange, both information from his doctor and other diabetic patients could help improve his health status. Prior studies have proved that social support can help decrease symptoms [26], our study provides further evidence on the whole health status and finds support on the role of social supports in improving the self-reported health status of diabetic patients. People with diabetes should be encouraged to actively engage in social exchange.

Third, we integrated the moderating effects of information price into the empirical model. Prior studies have examined the social exchange widely [10, 19, 20], however, they failed to consider the information cost. Consumers’ perceived cost is the core construct and foundation in an exchange [29] and maybe an impediment for consumers engaging in an exchange [30]. We conclude with two reasons, first, when people with diabetes have paid a higher price to obtain medical information, they may have a lower propensity to share relative information to others based on the transaction cost economics [31]. Second, if a diabetic patient still wants to buy services from doctors, it means that the current information does not provide enough knowledge to him. Then the information price is the cost to acquire and use a service. Based on our empirical results, we found that information price negatively moderates the relationship between information sharing from doctors and information sharing from people with diabetes. However, information price positively moderates the information exchange among people with diabetes. One possible explanation is that with the same disease, patients have sympathy for others with diabetes. Sharing information with others brings people with diabetes with the intrinsic benefit [21]. Therefore, when the information price is higher, it may inspire people with diabetes to feel a stronger sympathy and choose to share information to others publicly to help them. Moreover, when a consumer has paid a higher cost to get a service, he may hope to help others reduce their purchases by providing service related information.

### Implications

Our present study produces several insights, which have implications for social exchange, patient behavior, online health communities, and information technology in diabetes self-management literature.

Our study contributes to knowledge in several ways. First, our work extends our knowledge of the social exchange theory by integrating the role of information price. Prior studies believed that information exchange in virtual communities is a reciprocal process with maximizing benefits and minimizing costs [17], and widely explored consumers’ motivation to provide reviews online [10]. However, they considered opportunity cost and intrinsic cost [18] and failed to include the cost for getting the subject to exchange. Our study has revealed the significant role of information cost in information exchange behavior. Moreover, we found that the effects of information cost are heterogeneous, changing with exchange participants. Specifically, information cost enhances information exchange behaviors among patients and decreases information exchange behaviors between doctors and patients.

Second, our study enriches the knowledge of patient behavior on OHCs from the information exchange perspective. Prior studies on information exchange mainly focus on general online social media (e.g. Facebook and Twitter) [12, 32] and use survey design to gain results [14]. However, the information exchange on professional online medical platforms—online health communities has not yet received much research attention. Studies on patient behavior on OHCs mainly focus on patients’ purchase decision-making behavior [7, 33], the relationship between doctors’ service quality and patients’ review behavior [10], or only include one type of information exchange [13–16] and fail to consider information exchange among multi-participants in one study and investigate their integrated effects. Our study proved that information exchange behaviors are heterogeneous, they change with the exchange participants.

Third, our study provides insights resulting from exploring the effects of information exchange participation on the health status of people with diabetes and contributes to the role of information technology in improving health status and in diabetes self-management literature. Modern healthcare systems are designed to treat acute diseases rather than managing chronic diseases that require long-term care management [34]. Information technology applications perform an efficient and personalized follow-up of chronic diseases [35], including electronic health records [36], personal health records [37], mhealth [38], and other decision support systems [39]. However, they rarely focus on the role of OHCs in managing conditions of diabetes.

In practice, our findings offer insights for doctors, managers of OHCs, and health policymakers. We have verified that participating in online information exchange behavior has a positive effect on diabetic patients’ health status. We thus propose that health policymakers encourage hospitals and third-party platforms to promote information exchange by introducing incentives, especially for these chronic diseases. Our research findings provide health policymakers with insights on further promote the implementation of doctors’ multisite practice, and encourage doctors to contribute knowledge on online health communities.

Based on our findings, we propose that doctors could provide free text consultation services to the patients to promote the propensity of patients’ information sharing behavior. On the one hand, information from patients makes other patients more likely to understand the doctor and may facilitate the purchase. On the other hand, patients who actively engage in information exchange behavior have a better health status. In addition, doctors need to balance their time between free text consultation service and paid text consultation service.

We propose that the managers of OHCs should introduce more mechanisms to promote information sharing behavior, including both information sharing from doctors and information sharing from patients. The platform could give subsidies to doctors if they provide free services. If a patient gives feedback, the platform could give him a reward or a discount for purchasing the doctor’s service again later. Findings in our study provide managers of platforms with some guidelines on encouraging both doctors and patients to participate in information exchange.

### Limitations

Although this research has highlighted several notable findings and contributions, we acknowledge some limitations. First, our data is only collected from one OHC in China, future research could examine our results in other contexts, especially ones outside of China. Second, some variables in our study are measured by dummy variables, which may lead to small coefficients and fail to identify the clinically relevant effects. Future research could use more precise and complex methods to measure them. Third, we use self-reported health status to examine the consequence of information exchange behavior. Future research could use text mining to drug more information. In addition, other relevant dimensions of consequence can also be measured, such as health behaviors. Fourth, we only include free information sharing from doctors and feedbacks from diabetic patients, future research could measure other forms of information sharing.

## Conclusion

At present, urbanization, aging population, reduced physical activities, and increasing levels of overweight and obesity contribute to the increase of diabetes. OHCs, with a wealth of multi-source information exchange, have provided a convenient way for people with diabetes to actively participate in their self-management and have been widely used. This study is among the first that tests the information exchange behavior and consequence for people with diabetes in OHCs and examines the moderating effects of information price. The specific research questions being addressed in this paper include (a) does information sharing from a doctor affect a diabetic patient’s propensity to share information publicly, (b) does information sharing from other diabetic patients affect a diabetic patient’s propensity to share information publicly, (c) how does information price moderate the information exchange behaviors on OHCs, and (d) does information exchange in OHCs affect the health status of people with diabetes? Our present study produces several insights, which have implications for social exchange, patient behavior, online health communities, and information technology in diabetes self-management literature. By understanding the online information exchange behaviors of doctor-patient and patient-patient, we may be able to understand how to reach people to receive and deliver diabetes information through these professional OHCs.

## Data Availability

The datasets used and analyzed during the current study are available from the corresponding author on reasonable request.

## Abbreviation

OHCs: Online health communities
